# Prevalence of multidrug-resistant organisms in nursing homes in Belgium in 2015

**DOI:** 10.1371/journal.pone.0214327

**Published:** 2019-03-28

**Authors:** Katrien Latour, Te-Din Huang, Béatrice Jans, Catherine Berhin, Pierre Bogaerts, Audrey Noel, Claire Nonhoff, Magali Dodémont, Olivier Denis, Margareta Ieven, Katherine Loens, Didier Schoevaerdts, Boudewijn Catry, Youri Glupczynski

**Affiliations:** 1 Operational Directorate Epidemiology & Public Health, Sciensano, Brussels, Belgium; 2 Department of Public Health and Primary Care, University of Leuven, Leuven, Belgium; 3 National Reference Centre for antibiotic resistant Gram-negative bacilli, Laboratory of Clinical Microbiology, Centre hospitalier universitaire de Namur, Université catholique de Louvain, Yvoir, Belgium; 4 National Reference Centre for methicillin-resistant *Staphylococcus aureus* and staphylococci, Department of Clinical Microbiology, Hôpital Erasme, Université Libre de Bruxelles, Brussels, Belgium; 5 Ecole de Santé Publique, Université Libre de Bruxelles, Brussels, Belgium; 6 National Reference Centre for vancomycin-resistant enterococci, Department of Clinical Microbiology, University Hospital of Antwerp, Edegem, Belgium; 7 Department of Geriatric Medicine, Centre hospitalier universitaire de Namur, Université catholique de Louvain, Mont-Godinne, Belgium; 8 Institute of Health and Society, Ecole de Santé Publique, Université catholique de Louvain, Brussels, Belgium; Amphia Ziekenhuis, NETHERLANDS

## Abstract

**Objectives:**

Following two studies conducted in 2005 and 2011, a third prevalence survey of multidrug-resistant microorganisms (MDRO) was organised in Belgian nursing homes (NHs) using a similar methodology. The aim was to measure the prevalence of carriage of methicillin-resistant *Staphylococcus aureus* (MRSA), vancomycin-resistant enterococci (VRE), extended-spectrum β-lactamase producing *Enterobacteriaceae* (ESBLE) and carbapenemase-producing *Enterobacteriaceae* (CPE) in NH residents. Risk factors for MDRO carriage were also explored.

**Methods:**

Up to 51 randomly selected residents per NH were screened for MDRO carriage by trained local nurses between June and October 2015. Rectal swabs were cultured for ESBLE, CPE and VRE, while pooled samples of nose, throat and perineum and chronic wound swabs were obtained for culture of MRSA. Antimicrobial susceptibility testing, molecular detection of resistance genes and strain genotyping were performed. Significant risk factors for MDRO colonization MDRO was determined by univariate and multivariable analysis.

**Results:**

Overall, 1447 residents from 29 NHs were enrolled. The mean weighted prevalence of ESBLE and MRSA colonization was 11.3% and 9.0%, respectively. Co-colonization occurred in 1.8% of the residents. VRE and CPE carriage were identified in only one resident each. Impaired mobility and recent treatment with fluoroquinolones or with combinations of sulphonamides and trimethoprim were identified as risk factors for ESBLE carriage, while for MRSA these were previous MRSA carriage/infection, a stay in several different hospital wards during the past year, and a recent treatment with nitrofuran derivatives. Current antacid use was a predictor for both ESBL and MRSA carriage.

**Conclusions:**

In line with the evolution of MRSA and ESBL colonization/infection in hospitals, a decline in MRSA carriage and an increase in ESBLE prevalence was seen in Belgian NHs between 2005 and 2015. These results show that a systemic approach, including surveillance and enhancement of infection control and antimicrobial stewardship programs is needed in both acute and chronic care facilities.

## Introduction

The increasing prevalence of infections due to multidrug-resistant organisms (MDROs) represents a worldwide public health problem and is not only of importance in acute care hospitals [1]. Long-term care facilities (LTCFs), such as nursing homes (NHs), have been identified as important reservoirs of methicillin-resistant *Staphylococcus aureus* (MRSA) and extended-spectrum β-lactamase producing *Enterobacteriaceae* (ESBLE) by prevalence and incidence studies conducted in different European countries [2]. Recently, there have also been several reports of infection and/or colonization by other MDROs like carbapenemase-producing *Enterobacteriaceae* (CPE) and vancomycin-resistant enterococci (VRE) among its residents [3–15].

In many ways, LTCFs and NHs are favourable settings for the emergence and spread of antimicrobial resistance (AMR) over the healthcare network. These facilities outnumber acute care hospitals in numbers and total number of beds and although these facilities provide less specialized care and therefore have a lower daily probability of transmission of antimicrobial resistant pathogens, the spread can be more effective because of the much longer length of stay of the LTCF residents [16,17]. In addition, these facilities often strive to create a homelike environment. Infection prevention and control (IPC) guidelines are less stringent in NHs and adequately trained IPC personnel is often lacking [18,19]. Diagnostic uncertainty due to limited access to laboratory tests, sampling difficulties and atypical presentation of illness in frail older adults can lead to overuse of antibiotics and prolonged empirical therapy, which in turn potentially increase selection pressure on bacteria to become resistant [16,18,20]. Last but not least, frequent transfers of infected or colonized patients can lead to the diffusion of MDROs inside of and between acute care facilities and LTCFs [16,17,21].

Two cross-sectional surveys conducted in 2005 and 2011 in 60 Belgian different NHs showed that 19.5% and 12.2% of the screened residents were asymptomatic carriers of MRSA, respectively [22,23]. The 2011 survey also found that 6.2% of the residents were colonized with ESBLE and that none carried VRE. Carriage of CPE was at that time not explored [23].

At the end of 2011, the Superior Health Council of Belgium published an advice for the detection, prevention and control of CPE in Belgium, soon after the National Reference Centre for antibiotic resistant Gram-negative bacilli had noticed a rise in the number of cases [24]. A national surveillance program, combining epidemiological and microbiological data, was set up and results showed a significant increase in the number of CPE cases reported by different hospital laboratories. CPE strains were no longer only found in patients repatriated from abroad, but also in patients without any travel history and most were admitted to geriatric departments [25]. An American study identified transfer from high-acuity LTCFs to be associated with carriage of CPE upon hospital admission [26]. Although nursing homes in Belgium are less medicalized and serve as a home-like environment where residents in general stay until their end of life, we were interested in knowing the prevalence of CPE carriage in these facilities. Using a methodology comparable to the 2011 survey, we conducted a national prevalence study in 2015 to explore the asymptomatic carriage rate of CPE in addition to MRSA, ESBLE, and VRE.

The aim of the present paper is to present the prevalence of asymptomatic carriage of MRSA, ESBLE, CPE, and VRE found during the 2015 survey in Belgian NH residents. Moreover, risk factors associated with carriage of these MDRO are described.

## Materials and methods

### Study design

A cross-sectional prevalence survey was organised between June and October 2015. Thirty NHs (and two reserve facilities per selected NH) were systematically selected from the national insurance database, which was sorted according to region, NH size and proportion of high-care beds. An invitation to participate in the study was sent by regular mail to the director and coordinating physician of these facilities and all NHs were contacted by phone. In case of refusal of the primary selected NHs and its two substitutions, a NH from outside the initial selection was recruited by phone (active/selected recruitment). Preference was given to NHs with characteristics as close as possible to the primary selected NH, i.e. same regional distribution, NH size and proportion of high-care beds.

According to a previously described methodology the study coordinating team selected at random up to 51 residents (and 10 reserve) in each participating NH [22,23]. These residents had to be screened for MDRO carriage on one single day. In addition, if applicable, all roommates of selected residents had to be screened as well.

### Data collection

The study within the NH was coordinated by a local reference nurse and/or by the coordinating physician of the facility. This local surveyor had to complete a questionnaire for each participating resident. The questionnaire was similar to the form used during the 2011 survey and collected among others demographic (age, gender), length of stay in the NH, autonomy in the activities of daily living going from ‘less dependent’ (category O, A, B) to ‘highly dependent’ (category C, CD, D), mobility (ambulant, wheelchair-bound or bedridden), incontinence (for urine and/or faeces), disorientation in time and/or space, presence of pressure sores or other wounds, indwelling urinary catheter use, vascular catheter use, recent surgery (last 3 months), antacid use (proton pump inhibitors, H2 antihistamines), previously known MRSA, ESBLE, CPE, VRE carriage/infection (past 12 months), current and previous (past 3 months) antibiotic use and hospital stay during the past 12 months [23]. Comorbidity was evaluated using the Charlson’s Comorbidity Index and categorized in three groups (none or mild, moderate, severe) [27].

### Microbiological analysis

In each participating facility, trained local nurses performed a same-day series of sampling on all selected residents including: (a) a collection kit containing one tube and three application swabs for pooled sampling of nose, throat and perineum and a separate swab for a chronic skin wound (when present) for MRSA detection (eSwab with trypticase soy broth (TSB) enrichment broth + 2.5% NaCl, Copan, Brescia, Italy) and (b) a rectal swab for the detection of ESBLE, CPE and VRE. Rectal swab sampling was carried out by using transport swabs in Amies medium (Copan, Brescia, Italy). Specimens were refrigerated at 4°C for a maximum of 48h and analysed in a central laboratory according to a previously established protocol [23]. For MRSA, enriched broth swabs were streaked onto a selective chromogenic medium (MRSA-Select, Bio-Rad, Marnes-la-Coquette, France). Rectal swabs collected for ESBLE, CPE and VRE were cultured on group-specific selective chromogenic agars (chromID ESBL, chromID CARBA, chromID OXA-48, chromID VRE, bioMérieux, France) and on a MacConkey-medium (bioMérieux, Marcy L’Etoile, France) taken as a sampling quality control. Rectal swab specimens which did not yield any bacterial growth on the MacConkey agar were excluded from further analysis as they were considered of poor quality. Matrix-assisted laser desorption/ionization time-of-flight mass spectrometry (MALDI-TOF MS) using Microflex LT (Bruker Daltonics, Germany) based on the MALDI BioTyper database (version IVD 2.2 DB-5989 MSP) was used for bacterial identification of suspected colonies at species level.

#### Staphylococcus aureus

All isolates were submitted to a triplex polymerase chain reaction (PCR) assay targeting 16S rRNA-*mec*A-nuc and 16S-*mec*C PCR. All confirmed MRSA strains (one isolate per resident) were genotyped by *spa* typing and staphylococcal cassette chromosome *mec* (SCC*mec*) by determination of *ccr* and *mec* complexes. Multilocus Sequence Typing (MLST) clonal complexes (CCS) were inferred from *spa*-types from previously conducted surveys [28].

#### Enterobacteriaceae

All isolates of *Enterobacteriaceae* cultured on selective media were subjected to antibiotic susceptibility testing (AST; including cefotaxime, ceftazidime, cefepime, aztreonam, ertapenem, meropenem, ciprofloxacin, cotrimoxazole, amikacin and gentamicin). AST was performed by disc diffusion method according to the recommendations of the Clinical Laboratory Standards Institute (CLSI) [29]. ESBL production was confirmed by double disc combination synergy test, and carbapenemase production by hydrolysis-based Carba NP test [30]. Genotypic characterisation of resistance determinants was performed by multiplex PCR assays and by DNA microarray (CT103; Check-Points, Netherlands) [31]. Clonality among ESBL-positive *K*. *pneumoniae* strains (one isolate per resident) was assessed by Fourier transform infrared (FTIR) spectroscopy using the IR Biotyper system (IRB; Bruker Daltonik GmbH, Bremen, Germany). FTIR spectra were acquired following manufacturer’s instructions and clustered as IRB-types with the average linkage algorithm using the IR Biotyper software version 2.0 [32].

#### Enterococci

The susceptibility of all enterococcal isolates was performed by the Etest (bioMérieux, France). Interpretation was done according to the recommendation of the European Committee on Antimicrobial Susceptibility Testing (EUCAST) [33]. Multiplex PCR was used for detection of *vanA* and *vanB* genes. Isolates were typed using MLST techniques.

### Statistical analysis

Taking into account a cluster effect and an alpha level of 0.05, a sample size of 1530 residents was initially calculated to achieve an absolute precision of estimate of ± 1% with a confidence level of 95% and an expected prevalence of 12% for MRSA, 6% for ESBLE and 0.5% for both VRE and CPE.

Data were analysed using STATA 14.2 SE (StataCorp LP, Texas, USA). Median and interquartile ranges (IQRs) were calculated for continuous variables. Prevalence of MDRO carriage was calculated for each MDRO (number of residents with MDRO per 100 screened residents). The calculated prevalence rates were weighted, taking into account the number of residents actually tested in each NH, compared to the theoretical number of residents to test in each NH in the study. Poisson distribution was used to calculate the 95% confidence intervals (95%CI).

In order to explore risk factors of MDRO carriage, odds ratios (OR) and 95%CI were calculated using logistic regression analysis. All predictors with *p*-value < 0.10 in univariate analysis were included in multiple logistic regression models with stepwise backward elimination of the least significant variable until all remaining variables had *p*-value < 0.05.

### Ethics statement

The Ethical Committee of the University Hospital Centre of Namur (CHU UCL Namur; national number B039201523615) approved the protocol of this study. The local surveyors were responsible for seeking written informed consent from all residents participating in the study. If the health professional judged that a resident was incapable of consenting (e.g. in case of cognitive impairment), consent was obtained from his/her legal representative. All data were coded in order to protect the identification of residents and NHs. Positive microbiological results were reported to the general practitioners of the concerned residents.

## Results

### Participating nursing homes and residents

Twenty of the systematically selected NHs accepted to participate, i.e. nine primary selected NHs and 11 reserve facilities. The remaining ten NHs were subsequently recruited taking into account regional distribution, NH size and proportion of high-care beds (active/selected recruitment). Of these NHs, one did not complete the study and was excluded from further analysis.

In total, 1448 residents in 29 participating NHs (Flanders: [n = 16], Brussels: [n = 2], Walloon region: [n = 11]) were screened for MDRO carriage. Questionnaires were completed for 1441 of 1448 screened residents. The median age of the residents was 86 years (IQR: 81–91 years) and their median length of residency in the facility was 29 months (IQR: 12–60 months). Detailed characteristics of the study population are shown in Table 1.

**Table 1:** Characteristics of residents (n = 1441) included in a point prevalence survey conducted in 29 Belgian nursing homes, 2015.

Characteristics	Subcategory	Result*
Age, in years; median (IQR) [Range]		86 (81–91) [35–109]
Female/male gender, n (%)		1084 (75.5) / 351 (24.5)
LOS of the NH residents, in months; median (IQR) [Range]		29 (12–60) [0–443]
Number of residents in a single bed room, n (%)		1175 (81.8)
Level of autonomy according to the modified KATZ scale**, n (%)		
	Category O	113 (8.0)
	Category A	211 (14.8)
	Category B	402 (28.3)
	Category C/CD/D	696 (48.9)
Mobility level, n (%)		
	Ambulant	753 (54.6)
	Wheelchair/bedridden	626 (45.4)
Urinary and/or faecal incontinence, n (%)		842 (62.1)
Charlson’s Comorbidity Index, n (%)		
	None or mild	409 (31.5)
	Moderate	684 (52.7)
	Severe	204 (15.7)
Previous hospitalization in the year prior to the survey, n (%)		415 (28.9)
Previous stay in several different hospital wards in the year prior to the survey, n (%)		17 (1.2)
Known dementia, n (%)		702 (54.1)
Known chronic obstructive pulmonary disease, n (%)		152 (11.7)
Known recurrent urinary tract infection, n (%)		109 (8.4)
Previously known MRSA colonization (past year), n (%)		42 (2.9)
Previously known MRSA colonization/infection (past year), n (%)		48 (3.3)
Current MRSA decolonisation procedure at the time of survey, n (%)		12 (0.8)
Previously known ESBLE colonization (past year), n (%)		23 (1.6)
Previously known ESBLE colonization/infection (past year), n (%)		40 (2.8)
Previously known CPE colonization/infection (past year), n (%)		2 (0.1)
Previously known VRE colonization/infection (past year), n (%)		0 (0.0)
Wounds (pressure sores/ulcers, other wounds: surgical/traumatic), n (%)		117 (8.2)
Urinary catheter, n (%)		26 (1.8)
Vascular catheter, n (%)		2 (0.1)
Gastrostomy, n (%)		18 (1.3)
Tracheostomy, n (%)		2 (0.1)
Naso-gastric tube feeding, n (%)		12 (0.9)
Residents with current antibiotic use (the day of the survey), n (%)		73 (5.1)
Residents with previous antibiotic use (past 3 months), n (%)		323 (22.4)
Total number of antibacterials prescribed for systemic use [J01] in the past 3 months, n (%)		435 (100.0)
	Tetracyclines (J01A)	15 (3.4)
	Amphenicols (J01B)	2 (0.5)
	Beta-lactam penicillins (J01C)	139 (32.0)
	Other beta-lactams (J01D)	24 (5.5)
	Sulfonamides and trimethoprim (J01E)	18 (4.1)
	Macrolides, lincosamins and streptogrammins (J01F)	27 (6.2)
	Quinolones (J01M)	85 (19.5)
	Other antimicrobials (J01X)	125 (28.7)
Antacid use (proton pump inhibitors, H2 antihistamines) at the time of screening, n (%)		622 (45.3)

NH, nursing home; LOS, length of stay; IQR, interquartile range; MRSA, methicillin-resistant *Staphylococcus aureus;* ESBLE, extended-spectrum β-lactamase-producing *Enterobacteriaceae*; VRE, vancomycin-resistant enterococci; CPE, carbapenemase-producing *Enterobacteriaceae*.

*Missing values were not included in the percentage calculation

**Category O = complete autonomy and Category C, CD or D = highest level of dependency

The general characteristics of actively recruited NHs did not significantly differ from the randomly selected facilities (mean NH size: 114 beds versus 102 beds, proportion of high care beds: 56.3% versus 58.3%).

### Prevalence and microbiology of colonization

MDRO carriers were found in all participating facilities. In total, 1447 residents were screened for MRSA by pooled specimens (nose/throat/perineum). In addition, samples from wound lesions were obtained in 75 subjects (5.2%). MRSA carriage was identified in 133 residents. The weighted mean prevalence was 9.0% [95%CI: 8.1–10.3] and ranged from 0.0% in one NH to 21.6%.

Most isolates (n = 108, 81.8%) belonged to four hospital-associated lineages that are endemic in Belgium: CC-45-SCC*mec* type IV (n = 43); CC8-SCC*mec* type IV (n = 27); CC5-SCC*mec* type II (n = 23) and CC5-SCCmec type IV (n = 15) found in 18 (62.1%), 11 (37.9%), 5 (17.2%), 10 (34.5%) NHs, respectively (Table 2) [28]. Two MRSA isolates carried TSST-1, while no PVL-positive isolates were detected. Two livestock-associated MRSA strains (t011; CC-398) were isolated in NHs from the northern part of Belgium.

**Table 2:** Molecular typing of methicillin-resistant *Staphylococcus aureus* (MRSA) isolates (n = 132) in 29 Belgian nursing homes, 2015.

CC-SCC*mec*	Most frequent *spa*-type(s)	Number of isolates
CC5-II	t003	23
CC5-IV	t002	13
CC5-IV (2&5)	t002	2
CC5-VI	t777	12
CC8-IV	t008	23
CC8-IV (2&5)	t008, t2054	4
CC8-V	t008	1
CC22-IV	t032	3
CC45-II	t038	1
CC45-IV	t740	43^a^
CC45-NT	t330	1
CC398-V	t011	2
Others (IV or V)	various	4^a^

NT, SCC*mec* non-typeable.

^a^One isolate within this group carried toxic shock syndrome toxin 1 (TSST-1).

Twenty-five rectal swabs (1.7%) taken for screening of ESBLE, CPE and VRE carriage were excluded because of poor sampling quality. Among the remaining 1423 screened residents, 164 were found to carry one ESBLE, while four residents carried two. The weighted mean prevalence of ESBLE carriage was 11.3% [95%CI: 10.6–13.1], ranging from 0.0% in two NHs to 45.8%.

**Fig 1:**
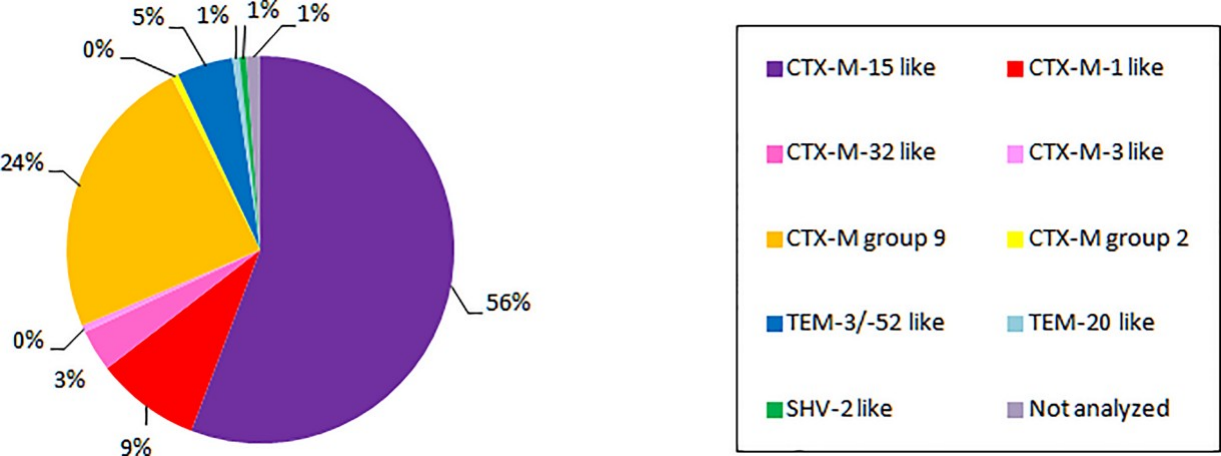
Distribution of ESBL-producing *Enterobacteriaceae* by type of enzyme (n = 172 isolates).

*Escherichia coli* was the most frequently isolated ESBL-positive *Enterobacteriaceae* species (n = 143, 83.1%), followed by *Klebsiella pneumoniae* (n = 29, 16.9%). The most predominant ESBL coding genes belonged to the CTX-M family (93.0%) with a predominance of CTX-M group 1 and especially of CTX-M-15 within this group (Fig 1).

The 143 ESBL-producing *E*. *coli* strains were widely distributed in 27 of the 29 participating NHs with CTX-M group 1 (n = 67; 46.9%) and CTX-M group 9 (n = 41; 28.7%) as the predominant ESBL enzymes found in 22 and 12 NHs, respectively.

All ESBL-positive *K*. *pneumoniae* strains harbored a CTX-M-15-like encoding gene. In two NHs, CTX-M-15-like ESBL-producing *K*. *pneumoniae* represented more than half of all isolated ESBLE strains (n = 10/17 in one NH and n = 8/15 in the other NH). Among these CTX-M-15-like ESBL *K*. *pneumoniae*, FTIR spectroscopy clustered 9/10 isolates of the first NH in the same partition and 7/8 isolates of the second NH in a different (unrelated) partition. The isolates from the other NHs were determined as different IRB-types.

The co-resistance rate to ciprofloxacin and co-trimoxazole was nearly 100% in ESBL-positive *K*. *pneumoniae* and overall lower but still substantial in ESBL-positive *E*. *coli* (74.8% (n = 107/143) resistant to ciprofloxacin and 42.0% (n = 60/143) to co-trimoxazole). A higher co-resistance rate was associated with CTX-M type ESBL producers (88.1% (n = 141/160) resistant to ciprofloxacin and/or co-trimoxazole) compared to other (TEM or SHV) types of ESBLs (40.0% (n = 4/10) resistant) (Table 3).

**Table 3:** Distribution of co-resistance to ciprofloxacin and/or co-trimoxazole among extended spectrum beta-lactame (ESBL) producing *Escherichia coli* and *Klebsiella pneumoniae* isolates in 29 Belgian nursing homes, 2015.

		*E*. *coli*			*K*. *pneumoniae*	Total
Ciprofloxacin	Co-trimoxazole	CTX-M type	Other type (TEM or SHV)	Not analyzed	CTX-M type	
Resistant	Resistant	47	1	1	28	77
	Susceptible	56	1	1	1	59
Susceptible	Resistant	9	2			11
	Susceptible	19	6			25
**Total**		**131**	**10**	**2**	**29**	**172**

The rate of co-colonization by MRSA and ESBLE was low (n = 27/1423; weighted mean prevalence 1.8% [95%CI: 1.4–2.5], ranging between 0 and 13.7%). There was no significant correlation between the prevalence of MRSA and ESBLE carriage in the participating NHs (p = 0.21).

The prevalence of CPE and of VRE carriage was low (less than 0.1%). One resident carried an OXA-48 carbapenemase-producing *K*. *pneumoniae* (along with a CTX-M-15-like ESBL). Additionally, non carbapenemase-producing carbapenem resistant *Enterobacteriaceae* (*Enterobacter cloacace* [n = 5], *K*. *pneumoniae* [n = 3] and *E*. *coli* [n = 1]) species were isolated from nine residents (0.6%). Only one resident carried a VRE isolate, subtyped as *vanA*-producing *Enterococcus faecium* (ST19).

In order to assess the occurrence of potential recruitment bias, we compared the observed prevalence rates according to the method of recruitment (20 randomly selected NHs versus the 9 actively recruited NHs). The prevalence of MRSA-carriers was not statistically different between the two cohorts (randomly selected NHs: 8.3% [95%CI: 7.1–9.7] versus actively recruited NHs: 11.0% [95%CI: 8.9–13.4], p = 0.10). On the other hand, the prevalence of ESBLE carriage was significantly higher in the group of actively recruited NHS (14.8% [95%CI: 12.4–17.5] compared to the randomly selected NHs (10.4% [95%CI: 9.1–12.0], p = 0.02).

### Risk factor analysis

Significant risk factors of MRSA and ESBLE carriage in univariate analysis (p<0.05) are presented in Table 4.

**Table 4:** Risk factors for colonization with MRSA and ESBLE among a random sample of residents screened within 29 Belgian nursing homes, 2015: Results from univariate analysis.

Predictors^a^	MRSA carriers (n = 133/1447)	ESBLE carriers (n = 168/1423)
	Unadjusted OR (95%CI); *p-*value	Unadjusted OR (95%CI); *p-*value
Gender (male)		0.67 (0.44–1.01); 0.055
Modified Katz score C, CD or D (highly dependent)		**1.70 (1.10–2.62); 0.002**
Mobility (wheelchair bound or bedridden)		**1.76 (1.26–2.46); 0.001**
Urinary and/or faecal incontinence		**1.54 (1.06–2.23); 0.022**
Pressure sore or skin ulcer		**2.35 (1.27–4.36); 0.007**
Recurrent urinary tract infections		**1.74 (1.04–2.92); 0.035**
Antacid use (proton pump inhibitors, H2 antihistamines)	**1.57 (1.09–2.26); 0.016**	**1.90 (1.35–2.66); <0.001**
Previously known MRSA carriage/infection*	**4.15 (2.18–7.92); <0.001**	
Previously known ESBLE carriage/infection*	**2.55 (1.15–5.65); 0.021**	
Previous antibiotic use (past 3 months)		**2.14 (1.52–3.02); <0.001**
≥ 3 antibiotics in the past 3 months		**3.69 (1.57–8.69); 0.003**
Previous antibiotic use with**:		
Penicillins with extended spectrum (J01CA)		**2.31 (1.03–5.16); 0.042**
Beta-lactamase resistant penicillins (J01CF)		5.10 (0.85–30.72); 0.076
Combinations of sulphonamides and trimethoprim, including derivatives (J01EE)		**6.10 (2.24–16.61); <0.001**
Fluoroquinolones (J01MA)	1.81 (0.95–3.43); 0.071	**2.73 (1.60–4.66); <0.001**
Nitrofuran derivatives (J01XE)	**2.53 (1.27–5.02); 0.008**	
Other antibacterials (J01XX)		**2.19 (1.03–4.68); 0.042**
Hospital stay (last year) in several different hospital wards	**4.22 (1.05–16.95); 0.008**	

MRSA, methicillin-resistant *Staphylococcus aureus;* ESBLE, extended-spectrum β-lactamase-producing *Enterobacteriaceae*

^a^ Only predictors with a *p*-value < 0.10 in univariate analysis are reported in this table.

* ‘*previous known MRSA or ESBLE carriage/infection’* = the resident has antecedents of MRSA or ESBLE carriage/infection (past year).

** Classification according to WHO ATC system (http://www.whocc.no/atc_ddd_index/)

Using multiple logistic regression (Table 5), the best predictors of being colonized by ESBLE were impaired mobility (being wheelchair bound or bedridden), recent antimicrobial treatment (within the past 3 months) with fluoroquinolones (J01MA) or with combinations of sulphonamides and trimethoprim, including derivatives (J01EE) and ongoing use of antacids (proton pump inhibitors, H2 antihistamines). The latter was also found to be a predictor of MRSA carriage, in addition to previously known carriage/infection with MRSA (past 12 months), use of nitrofuran derivatives (J01XE) in the past 3 months and a stay in several different hospital wards in the past year.

**Table 5:** Multiple logistic regression analysis of individual risk factors for carriage of MRSA (3A), of ESBLE (3B) among a random sample of residents screened within 29 Belgian nursing homes, 2015.

**3A: Predictors for MRSA carriage**	**Adjusted OR (95%CI)**	***p-*value**	
Previously known MRSA carriage/infection (past 12 months)	3.77 (1.91–7.45)	<0.001	
Previous use of nitrofuran derivatives (J01XE; past 3 months)	2.25 (1.11–4.55)	0.024	
Recent stay in several different hospital wards (past 12 months)	3.99 (1.24–12.81)	0.020	
Current antacid use (proton pump inhibitors, H2 antihistamines)	1.49 (1.03–2.16)	0.034	
**3B: Predictors for ESBLE carriage**	**Adjusted OR (95%CI)**		***p*-value**
Mobility (wheelchair bound or bedridden)	1.60 (1.11–2.30)		0.011
Previous use of fluoroquinolones (J01MA; past 3 months)	2.60 (1.48–4.55)		0.001
Previous use of combinations of sulphonamides and trimethoprim, including derivatives (J01EE; past 3 months)	5.40 (1.94–15.07)		0.001
Current antacid use (proton pump inhibitors, H2 antihistamines)	1.74 (1.22–2.48)		0.002

MRSA = methicillin-resistant *Staphylococcus aureus;* ESBLE = extended-spectrum β-lactamase-producing *Enterobacteriaceae*

## Discussion

In this multicentre study, the mean weighted prevalence of asymptomatic MRSA and ESBLE carriage among NH residents was 9.0% and 11.3%, respectively. Only one CPE and one VRE carrier were found (prevalence less than 0.1%).

Over the past decades, several studies (mainly point prevalence surveys) have been conducted to explore MDRO carriage/infection in European LTCFs [2]. When interpreting and comparing the results and outcomes of these studies, not only methodological variabilities (e.g. differences in applied microbiological methods, in screened sampling sites or in criteria for the focused MDROs) should be considered but also selection criteria such as the type of LTCF in which the study is conducted. LTCFs represent a very large scope of facilities (from residential care to sub-acute LTCFs), with important variations in medical and social services provided, length of stay, population case-mix, organizational structure and available resources [34]. In addition to the variations in the epidemiology of antimicrobial resistance, differences in methodology and study setting can probably also account in part for the large inter-country variations.

For the third time in Belgium, we explored asymptomatic MRSA carriage among NH residents using a comparable methodology. The mean weighted prevalence of asymptomatic MRSA carriage significantly decreased between 2005 (19.5%; 95%CI: 16.4–21.5) and 2011 (12.2%; 95%CI: 11.3–13.1) and continued to drop to 9.0% (95%: 8.1–10.3) in 2015 [22,23]. A similar downward trend was observed in the mandatory surveillance of MRSA in acute care hospitals in Belgium where the incidence decreased from 4.0 hospital-acquired MRSA cases per 1000 admissions in 2003 to 1.1 cases per 1000 admissions in 2015 (annual decrease of 0.23 cases per 1000 admission, p<0.001) [35]. Since the late 1990s, several initiatives have been implemented in order to limit the spread of MRSA in healthcare facilities in Belgium, including the publication and updates of national guidelines for the control of transmission of MRSA in acute and chronic care sectors [36–39], repeated national campaigns in order to promote hand hygiene in hospitals [40,41] and national initiatives in order to rationalize antimicrobial use both in acute care and in the community [42,43].

The implementation of all these multifaceted interventions contributed to the decrease in the incidence of MRSA among hospitalized patients as well to the decrease in the prevalence of MRSA carriage in NH residents, but has not been successful in curbing the evolution of multidrug resistance in Gram-negative bacteria in Belgian care facilities. The prevalence of asymptomatic ESBLE carriage among NH residents almost doubled between 2011 and 2015, i.e. 6.2% (95%CI: 5.6–6.9) to 11.3% (95%CI: 10.6–13.1). In parallel, the surveillance of multidrug resistant Gram-negative bacteria in Belgian acute care hospitals showed a steadily increasing incidence of ESBLE colonization and infection over the last decade: from 2.2 cases per 1000 admissions in 2005 to 7.1 cases in 2015 for *E*. *coli* and from 0.7 to 3.2 cases per 1000 admissions for *K*. *pneumonia* [35]. In both healthcare facility types, *E*. *coli* remains the most frequently encountered ESBLE, but the proportion of *K*. *pneumoniae* among ESBLE considerably increased between 2011 (n = 10/205; 4.9%) and 2015 (n = 29/172; 16.9%) in NHs [23]. This constitutes an alarming evolution owing to the subsequent risk for the development of invasive infections in colonized patients and the higher potential of *K*. *pneumoniae* to cause outbreaks in hospitals and NHs.

Regarding the principal ESBL types circulating in the participating NHs, our results are in line with previous studies conducted in Belgium and in other European countries indicating that the CTX-M types, mostly CTX-M group 1 (and with CTX-M-15 largely predominant within this group), have continued to disseminate widely in *E*. *coli* and in *K*. *pneumoniae* species and are now almost completely replacing the SHV- and TEM-type ESBLs [44,45]. In the two NHs with high prevalence of CTX-M-15-like-producing *K*. *pneumoniae*, the identification of two independent clusters confirmed by typing with MALDI-TOF FTIR spectroscopy strongly suggested local ESBLE clonal cross-transmission. Although an important ESBLE inflow from a nearby acute care hospital was reported in one of these two NHs (KL, *personal communication with the concerning hospital and NH*), the occurrence of local transmission is also supported by the fact that several of the carriers were not recently hospitalized.

While in the past five years, nosocomial VRE and CPE outbreaks have been reported in several Belgian acute care hospitals, our study showed very low numbers of NH residents colonized by these MDROs [23]. The same was observed in another prevalence study which found no CPE colonization in Belgian NH residents [46]. A possible explanation for the low CPE and VRE colonization rates might be the overall lower use of antimicrobial agents in our NHs. In the present study, 5.1% of the participating residents received an antimicrobial on the day of the survey. This prevalence is significantly lower than the 28.9% found in the point prevalence study of healthcare-associated infections and antimicrobial use conducted in 2011 in Belgian acute care hospitals [47]. Also the spectrum of antimicrobials prescribed in NHs is very different compared to acute care hospitals in Belgium. Parenterally administered drugs such as cephalosporins, carbapenems, glycopeptides or aminoglycosides are only being prescribed very infrequently in NHs residents [48].

In the present study, previous use (past 3 months) of nitrofuran derivatives was identified as a predictor for MRSA carriage, while previous use of fluoroquinolones and combinations of sulphonamides and trimethoprim were risk factors for ESBLE in the multivariable analysis. In 2005, MRSA carriage was linked to recent use of fluoroquinolone use and amoxicillin and clavulanic acid [22]. In 2011, recent antibiotic use in general was a risk factor for ESBLE carriage, but not for MRSA [23]. Several other studies also found ‘recent antimicrobial use’ as risk factor for MDRO carriage/infection in NHs [2,49–51].

In the literature, recent hospital stay is also frequently mentioned as a risk factor for MRSA carriage [2,52,53]. The same was found in the multivariable analysis of our present survey, especially when admitted in several different hospital wards, and in the 2005 study [22].

In Belgium, acute care hospitals and NHs seem to act as ‘communicating vessels’ at least for MRSA and for ESBLE as exemplified by the parallel increasing/decreasing trends and observed ESBL enzyme types and MRSA *spa* types in both types of care facilities [22]. Transmission of MDROs is facilitated by frequent transfers of patients/residents between chronic and acute care sectors. In the present study, 28.9% of the NH residents had been admitted to an acute care hospital at least once in the past 12 months.

Bed shortages and bed management strategies are leading to an increasing number of patient transfers between hospital wards. A recent study with retrospective analysis of hospital data showed that 10 000 patients were moved 34 715 times in one year which equates to an average of 2.4 transfers per patient [54]. During hospitalization, NH residents can be colonized with MDROs endemic in the different wards, facilitating the dissemination of these organisms when returning to the NH. Characteristics of the NH and its resident population can contribute to an increasing risk for colonization/infection with MDROs. Compared to acute care hospitals, NHs are ‘home replacing environments’ where residents have frequent social/care contacts with healthcare staff and other residents. Furthermore, the workload is often very important and NH staff generally have less experience in mastering infection control and (hand)hygiene practices [55,56]. NHs can become reservoirs, subsequently leading to an increasing MDRO inflow in acute care hospitals. In order to interrupt this vicious circle, an appropriate communication between facilities during transfer as well as implementation of well-coordinated screening policies are urgently needed in both acute and long-term care.

Colonization history by the same microorganism was identified as risk factor for both MRSA and ESBLE in different studies conducted across Europe [2]. In both the current study and the 2011 survey, previously known MRSA carriage/infection (past 12 months) was associated with higher risk for MRSA carriage at the time of the study. However, in contrast to 2011 a history of ESBLE carriage was no longer a predictor for ESBLE in the present study [23].

Physical disability and low functional status are often associated with an increased risk for the acquisition of MDROs as these residents require more nursing and/or medical care and therefore have more frequent contacts with healthcare workers [2,7]. Indeed, in this study we found impaired mobility to be associated with an increased risk for ESBLE carriage, but not for MRSA. This matches exactly to our findings in the 2011 study [23]. In the first national study (2005) impaired mobility was a significant risk factor for MRSA carriage [22].

In our previous study, we were surprised to see recent/current intake of gastric antacid agents (proton-pump inhibitors or anti-H2 blockers) as a significant risk factor for MRSA carriage [23]. Risk analysis in the current study again indicated antacid use as a risk factor for MRSA, but this time also for ESBLE. While the use of antacids is a well-known risk factor for *Clostridium difficile* associated disease, only few studies describe antacid use as a risk factor for ESBLE carriage in the community [57] and in the hospital setting [58–60]. A plausible explanation for the association between antacid use and ESBLE colonisation is the mechanism of a disrupted barrier due to an increased gastric pH (functionality of the gastric barrier) and therefore diminished defence system. Antacids such as PPIs are modifying the gut microbiota [61]. In a recent study, analysis of the gut microbiome composition in a large number of individuals revealed major differences in microbiota composition in PPI users versus non-users [62]. PPI use was associated with a significant decrease in Shannon's diversity and with changes in 20% of the bacterial taxa. In PPI users, the investigators observed a significant increase in bacteria: genera *Enterococcus*, *Streptococcus*, *Staphylococcus* and the potentially pathogenic species *E*. *coli*. The risk of antacid use is particularly important, because antacids are widely used in Belgian NHs (45.3% of all participating NH residents in this study).

Other risk factors such as a long length of study, history of invasive procedures/devices, pressure sore/ulcers and underlying pathologies/comorbidities that are frequently reported in the literature to be associated with MDRO carriage in NH residents, were not found in our study [2].

We do acknowledge that this study had several limitations. First, the study design (point prevalence survey) did not allow us to investigate more in detail the dynamics of MDRO colonization (acquisition, persistence and clearance of carriage). Therefore, follow-up studies would have been needed, but these investigations were no aims of our study. A second limitation is that roughly one third of the participating NHs were actively recruited, taking into account the geographic location, NH size and proportion of high-skilled beds from the randomly selected NHs they replaced. A higher prevalence of ESBL carriage was observed in the actively recruited NHs (14.8%) compared to the randomly selected NHs (10.4%, p = 0.02). The reasons accounting for the higher prevalence of ESBLE in this subgroup of institutions could not be elucidated. Further and in comparison to the previous two national surveys, we no longer presented data by region as we only aimed to have representative data at national level.

Despite these limitations, we believe this study is of interest because in contrast to most other European multicentre point prevalence studies it provided simultaneously data for carriage of MRSA, ESBLE, VRE and CPE in NHs. Since we used a comparable methodology throughout the three surveys, we have been able to closely monitor the evolution of MDRO carriage in our Belgian NHs over a decade. The data were also useful for increasing awareness and fostering the implementation of programs aiming to improve infection control and antimicrobial stewardship in LTCFs at national level. Moreover, the prevalence data and identified risk factors have been of added value for training of healthcare personnel at the NH level.

## Conclusions

The results of the present survey and of the two previous studies provided valuable insight in the evolution of MDRO in LTCFs. The decreasing trend in MRSA carriage and increasing trend in ESBLE carriage among our NH residents are in line with the evolution of the MRSA and ESBL surveillance data in Belgian acute care hospitals. This confirms that acute and chronic care facilities (including NHs) can act as communicating vessels and therefore require a ‘systemic’ approach. Improvement of communication and inter-facility transfer policies between these two sectors are urgently needed. Additional efforts to enhance the compliance to standard precautions including personal hygiene, hand hygiene and environmental hygiene as well as antimicrobial stewardship programs in NHs are needed in order to stop the acquisition and transmission of MDROs in healthcare facilities.
